# Pine Pollen Polysaccharides’ and Sulfated Polysaccharides’ Effects on UC Mice through Modulation of Cell Tight Junctions and RIPK3-Dependent Necroptosis Pathways

**DOI:** 10.3390/molecules27227682

**Published:** 2022-11-08

**Authors:** Zhenxiang Li, Hanyue Wang, Zhanjiang Wang, Yue Geng

**Affiliations:** Key Laboratory of Food Nutrition and Safety of SDNU, Provincial Key Laboratory of Animal Resistant Biology, College of Life Science, Shandong Normal University, Jinan 250014, China

**Keywords:** pine pollen polysaccharides, sulfated polysaccharides, ulcerative colitis, tight junction proteins, necroptosis

## Abstract

The purpose of this study is to explore the effects of pine pollen polysaccharides and sulfated polysaccharides on mice with ulcerative colitis and whether they could protect mice from inflammation by regulating the tight junctions of colonic epithelial cells and regulating the RIPK3-dependent necroptosis pathways. Pine pollen polysaccharides were prepared by water boiling and ethanol precipitation. After deproteinedization with trichloroacetic acid, the UV spectrum showed that there were no proteins. One polysaccharide component (PPM60-III) was made by gel filtration chromatography, and then sulfated polysaccharide (SPPM60-III) was derived using the chlorosulfonic acid-pyridine method. After treatment with PPM60-III and SPPM60-III, the body weight of mice with ulcerative colitis induced by dextran sodium sulfate increased, the DAI score decreased, the levels of pro-inflammatory factors and inflammation-related enzymes decreased, and the level of anti-inflammatory factors increased. In addition, after treatment, the expressions levels of tight junction proteins increased, the expressions levels of key proteins of programmed necroptosis decreased, while the level of Caspase-8 increased. The results indicated that pine pollen polysaccharides and sulfated polysaccharides have a certain therapeutic effect on UC mice, and the therapeutic effect may be achieved by regulating the tight junction of colonic epithelial cells and regulating the RIPK3-dependent necroptosis pathways

## 1. Introduction

*Pinus yunnanensis* is one of the main forest tree species distributed in the southwest of China [1]. Yunnan pine pollen is the dry pollen of *Pinus yunnanensis*. In addition to beauty, pine pollen has remarkable curative effects on eczema, prostatic hyperplasia, hypertension, prostatitis, anti-aging, anti-tumor, liver protection, hypoglycemic, and other diseases [2,3,4]. Sulfated polysaccharides are natural and semi-synthetic acidic polysaccharides containing sulfate groups. After the sulfated polysaccharide, it is easy to bind to the specific domain of protein, thus changing its conformation and affecting its biological activity [5].

Inflammatory bowel disease (IBD) is a chronic nonspecific inflammatory bowel disease, which is generally classified as ulcerative colitis (UC) and Crohn’ s disease (CD). There is no exact cause yet, and it could cause persistent and enlarged mucosal inflammation in the intestine, resulting in bloody diarrhea, colonic motor dysfunction and tissue damage, and rectal urgency. There is no exact cause of UC. There are 7.6 to 245 cases of UC per 100,000 people every year, and the incidence is still rising. About one-third of UC patients experience a 10-year course of disease [6]. At present, UC has become a global disease and exploring new effective drugs for the treatment of UC is still a top priority. Studies have shown that Chinese herbal medicine *Dendrobium huoshanense* polysaccharides could prevent the inflammatory response in rats with UC by inhibiting the NF-κB signaling pathway and reducing IL-1β, TNF-α, IL-17, and TGF-β levels [7]. A neutral polysaccharide from *Ophiocordyceps lanpingensis* attenuated the inflammatory response of RAW264.7 macrophages by increasing the expression of IL-10 [8].

The intestinal mucosal barrier (IMB) is the first barrier against harsh environments. It is composed of tight junctions between epithelial cells and a single layer of columnar epithelium [9]. The tight junction is composed of two proteins: transmembrane proteins (they can interact with adjacent endothelial cells, constituting a physical barrier to prevent the proliferation of cells, such as Occludin and Claudin-1) and the accessory proteins (anchoring transmembrane proteins to cytoskeleton, ZO family) [10]. It has been confirmed that the destruction of intestinal mucosal barrier, the reduction of cell tight junctions, and the loss of barrier integrity are considered to be the causes of inflammatory bowel disease in UC patients [11]. Polysaccharides play an important role in restoring the tight junction of cells to regulate inflammation. For example, *Angelica sinensis* polysaccharides could significantly improve the symptoms of weight loss, disease activity index score, and colon shortening caused by DSS. It could also significantly inhibit the activity of myeloperoxidase in colon tissue, reduce the production of pro-inflammatory factors, and increase the expressions of tight junction proteins, thereby improving the ulcerative colitis in mice [12]. *Scutellaria baicalensis* Georgi polysaccharide could increase the expressions of tight junction proteins ZO-1, Claudin-5, and Occludin and improve inflammatory injury in mice [13]. Several inflammatory cytokines, including TNF-α and IL-1β, have been shown to mediate epithelial cell damage and alter intestinal permeability by altering the expression of TJ proteins, while anti-inflammatory cytokines, such as IL-10, could block intestinal damage caused by bacterial infection [14,15,16,17].

Necroptosis is a programmed cell death mode different from apoptosis and traditional necrosis, which could be initiated by tumor necrosis factor receptor (TNFR) or pattern recognition receptor (PRR). Necroptosis is mediated by receptor-interacting protein kinase 1 (RIPK1), receptor-interacting protein kinase 3 (RIPK3), and mixed lineage kinase domain-like protein (MLKL) [18]. Fas-associated death domain protein (FADD), as an adapter, induces apoptosis by recruiting and activating Caspase-8. FADD deficiency leads to RIPK1 production and necroptosis. RIPK3 acts on the upstream and phosphorylates RIPK1, thereby mediating its autophosphorylation [19]. The kinase activities of RIPK3 and RIPK1 are particularly important for the stable formation of RIPK1–RIPK3 programmed necrosis complex [20]. MLKL is phosphorylated by RIPK3 at threonine 357 and serine 358 and the occurrence of these phosphorylations is crucial to the programmed necroptosis pathway [21]. Necroptosis is also particularly closely related to inflammation. In the case of apoptosis, the secretion of cytokines does not exist or is very small, and programmed necroptosis is the original event leading to inflammation. Cell release of damage-associated molecular patterns (DAMPs) is the main way that RIPK3 stimulates inflammation after MLKL insertion [22]. At present, scientists are exploring the relationship between necroptosis and UC. Hesperidin could significantly reduce DSS-induced colitis injury by increasing the expression of ZO-1 and reducing the expressions of inflammatory factors, RIPK3 and MLKL [23]. Dihydrotanshinone could alleviate DSS-induced UC mice by regulating necroptosis-related proteins RIPK1, RIPK3, and MLKL and reducing the expressions of COX-2 and iNOS [24].

Studies have shown that pine pollen polysaccharide can reduce the inflammatory response of ulcerative colitis mice by regulating intestinal flora [25]. Previous studies have confirmed that pine pollen polysaccharide and its esterified products have therapeutic effects on ulcerative colitis mice at the immune level. It is speculated whether the regulation of immune factors by pine pollen polysaccharide and esterified polysaccharide is related to the protection of intestinal epithelial cell barrier [26]. The purpose of this study was to explore the therapeutic effect of pine pollen polysaccharides and sulfated polysaccharides on UC mice and whether they affect ulcerative colitis in mice by regulating cells’ tight junction and regulating the RIPK3 pathway. The results were helpful for food research and development.

## 2. Results

### 2.1. Extraction, Separation, and Sulfation of Polysaccharides from Pollen of Pinus yunnanensis

**Figure 1:**
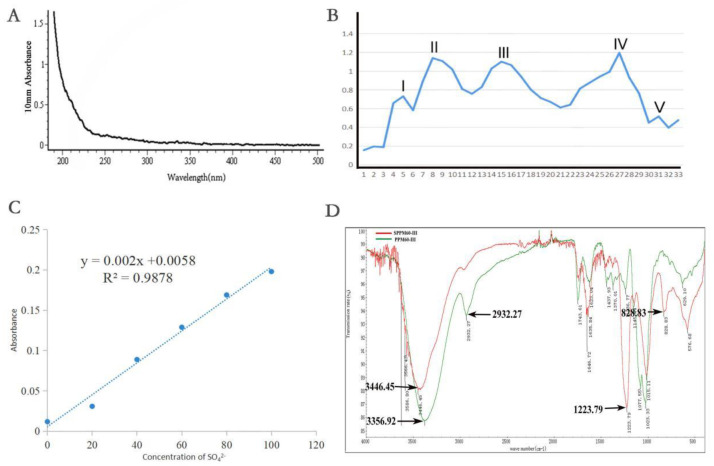
Extraction, separation and sulfation of polysaccharides from pollen of *Pinus yunnanensis*. (**A**) Ultraviolet spectrum; (**B**) the separation of the curve; (**C**) degree of substitution test; (**D**) infrared spectrum.

A total of 500 g of dried pollen of *Pinus yunnanensis* was used in the experiment. After water boiling and ethanol precipitation, deproteinization with trichloroacetic acid was carried out. Finally, the polysaccharides PPM40, PPM60, and PPM80 precipitated by different concentrations of ethanol were 2.280 g (0.456%), 7.730 g (1.546%), and 3.730 g (0.746%), respectively. The absorption peak of protein is about 260–280 nm. The results of the deproteinization experiments showed that there was no protein absorption peak, indicating that the protein removal was successful (Figure 1A). The results of polysaccharides’ separation and purification by Sephacryl S-400 HR showed that PPM60 was divided into five peaks, named I, II, III, IV, and V. The yield of the third component was the highest, so PPM60-III was selected for the experiment (Figure 1B). Here, 563 mg PPM60-III was obtained by multiple separation and purification and the yield was 20.11%. The standard curve equation of sulfate is y = 0.002x + 0.0058 (Figure 1C) and the degree of substitution of SPPM60-III is 1.28. It was composed of Gal/Glu/Xyl/Man/Rha and an unknown monosaccharide at the molar ratios 12.830:10.449:29.693:1:1.415:1.426 [26,27]. The IR results showed that PPM60-III showed a C-H stretching shock absorption peak at 2932.27 cm^-1^ and O-H absorption peak at 3356.92 cm^-1^, which were characteristic peaks of polysaccharides. At 3446.45 cm^-1^, SPPM60-III showed an O-H expansion peak, S=O characteristic absorption peak at 1223.79 cm^-1^, and C-O-S characteristic peak at 828.83 cm^-1^. Both polysaccharides contained O-H stretching shock absorption peaks, but the intensity of the absorption peak of SPPM60-III was weaker than that of PPM60-III, indicating that the -OH of PPM60-III was replaced by SO_4_^2-^ of SPPM60-III, indicating that the polysaccharides had been esterified successfully (Figure 1D).

### 2.2. Effects of PPM60-III and SPPM60-III on UC Mice

**Figure 2:**
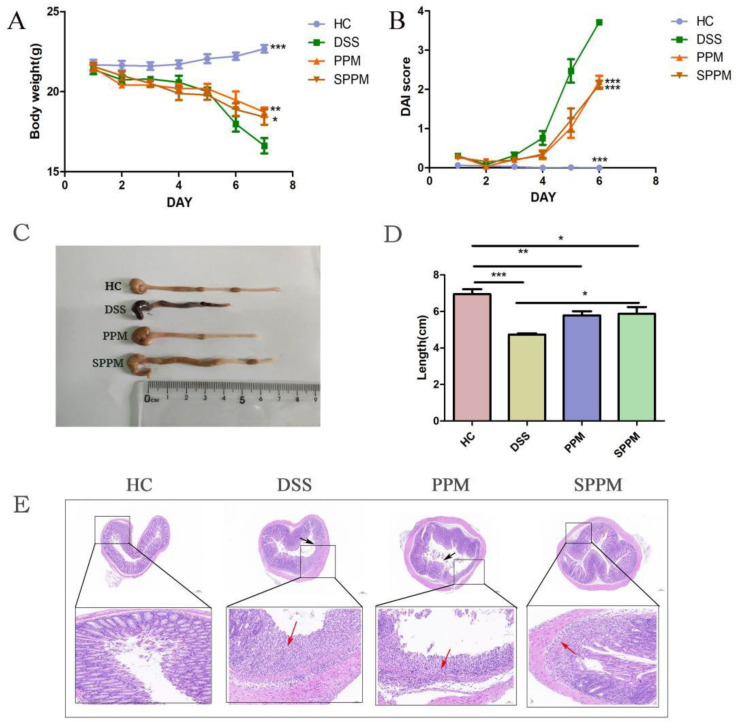
PPM60-III and SPPM60-III reduced inflammation in mice. (**A**) Body weight; (**B**) DAI score; (**C**) comparison of colonic length in mice; (**D**) length of mouse colon; and (**E**) HE staining of colon. Black arrows show shedding of epithelial cells and red arrows show infiltrating of inflammatory cells. * *p* < 0.05, ** *p* < 0.01, *** *p* < 0.001.

As shown in Figure 2A, the body weight of mice in the HC group showed a slow upward trend and, in the DSS group, the body weight decreased significantly, while the PPM and SPPM groups improved this situation. The DAI score in the DSS group rose sharply from day 4, while the PPM and SPPM groups alleviated the trend (Figure 2B). Compared with the HC group, the colon length of the DSS group was significantly shortened, while PPM60-III and SPPM60-III alleviated the shortening of colon length in mice (Figure 2C,D). HE staining showed thate thHC group had a large number of goblet cells and an intact mucus layer, while those goblet cells and mucus layer in the DSS group were seriously destroyed with the extensive infiltration of inflammatory cells. PPM60-III and SPPM60-III had a certain protective effect on the colon of mice and the SPPM group showed better improvement (Figure 2E). The results showed that PPM60-III and SPPM60-III had a certain protective effect on DSS-induced UC.

### 2.3. Effects of PPM60-III and SPPM60-III on Inflammatory Factors and Related Enzymes in UC Mice

**Figure 3:**
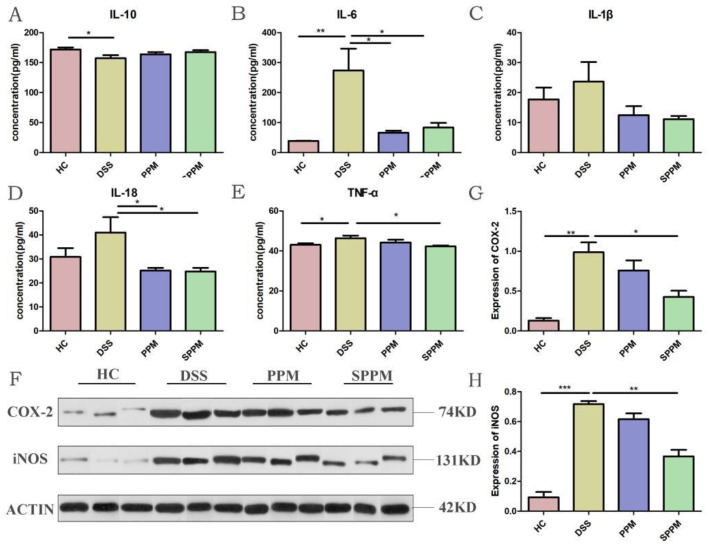
Effects of PPM60-III and SPPM60-III on inflammatory factors and related enzymes in mice. (**A**) The expression of IL-10; (**B**) the expression of IL-6; (**C**) the expression of IL-18; (**D**) the expression of IL-1β; (**E**) the expression of TNF-α; (**F**) Western blotting of Cox-2 and iNOS; (**G**) Cox-2 protein expression; (**H**) iNOS protein expression. * *p* < 0.05, ** *p* < 0.01, *** *p* < 0.001.

As shown in Figure 3A–E, compared with the HC group, the DSS group decreased the level of anti-inflammatory factor IL-10, while the PPM group and SPPM group could increase the level of IL-10, but there was no significant difference. On the contrary, the levels of pro-inflammatory factors IL-6, IL-18, IL-1β, and TNF-α in the DSS group were higher than those in the HC group. The levels of the PPM and SPPM groups were decreased to varying degrees and the regulation of IL-6 and IL-18 by PPM60-III and SPPM60-III was significant. These results indicated that PPM60-III and SPPM60-III could regulate inflammation by regulating the expression of inflammatory factors. Western blotting showed that PPM60-III and SPPM60-III could also reduce the expressions of inflammatory enzymes COX-2 and iNOS induced by DSS, and SPPM60-III had more significant changes in their content (*p* < 0.01) (Figure 3F–H). The above experimental results indicated that PPM60-III and SPPM60-III could regulate inflammation by regulating the expressions of inflammatory cytokines and related enzymes.

### 2.4. Effect of PPM6-III and SPPM6-III on Tight Junctions of Colonic Epithelium in UC Mice

**Figure 4:**
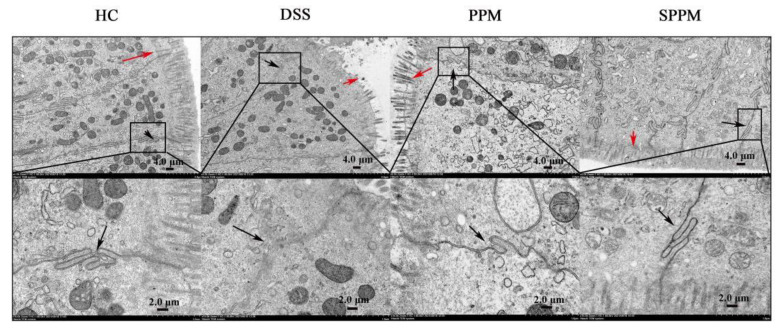
Transmission electron microscopy of tight junctions in mouse colonic tissue. The red arrow points to the microvilli on the epithelial surface of the colon mucosa and the black arrow points to the tight junctions of cells.

As shown in Figure 4, the results of transmission electron microscopy (TEM) showed that the microvilli on the epithelial surface of the colon mucosa of mice in HC group were complete and neatly arranged, with the same length and size. The epithelial cells were closely and clearly connected. In the DSS group, the microvilli on the epithelial surface of colonic mucosa were lost and the junctions between epithelial cells were blurred and loose. The intercellular connection in the PPM group was clearer than that in the DSS group and the loss of intestinal villi was slightly improved, but some of them still fell off. The microvilli of colonic epithelial cells in SPPM group were almost intact, arranged neatly, with the same size and length, and the cell connections were close and clear.

**Figure 5:**
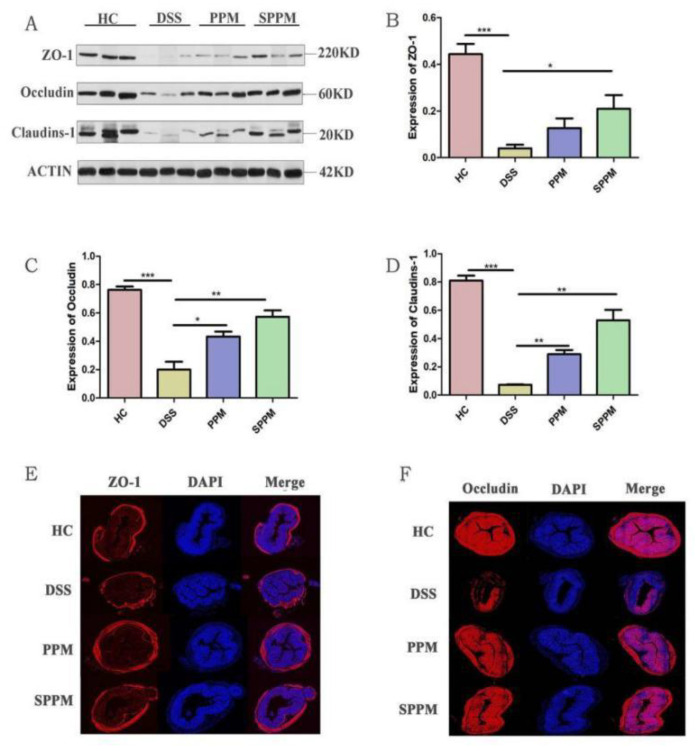
Effects of PPM60-III and SPPM60-III on tight junction protein in mice. (**A**) Western blotting; (**B**–**D**) protein expressions levels of ZO-1, occludin, and claudins-1; (**E**) ZO-1; (**F**) occludin. * *p* < 0.05, ** *p <* 0.01, *** *p* < 0.001.

The integrity of the intestinal mucosal barrier is maintained by tight junction proteins, including ZO-1, Occludin, and Claudin-1. Western blotting showed that the protein expressions of ZO-1, Occludin, and Claudin-1 in the DSS group were significantly decreased compared with the HC group (*p* < 0.001), while the protein expressions in the PPM and SPPM groups were restored to different degrees, among which the SPPM group had a better recovery effect and significantly increased the expressions of the ZO-1 (*p* < 0.05), Occludin (*p* < 0.01), and Claudin-1 (*p* < 0.01) proteins (Figure 5A–D). ZO-1 and Occludin immunofluorescence results were consistent with the Western blotting results (Figure 5E,F). The results showed that PPM60-III and SPPM60-III could restore the tight junctions of cells and regulate the expressions of tight junctions in cells to regulate inflammation, and SPPM60-III had a better effect on tight junctions.

### 2.5. Effects of PPM60-III and SPPM60-III on UC Mice by Regulating the RIPK3 Pathway

**Figure 6:**
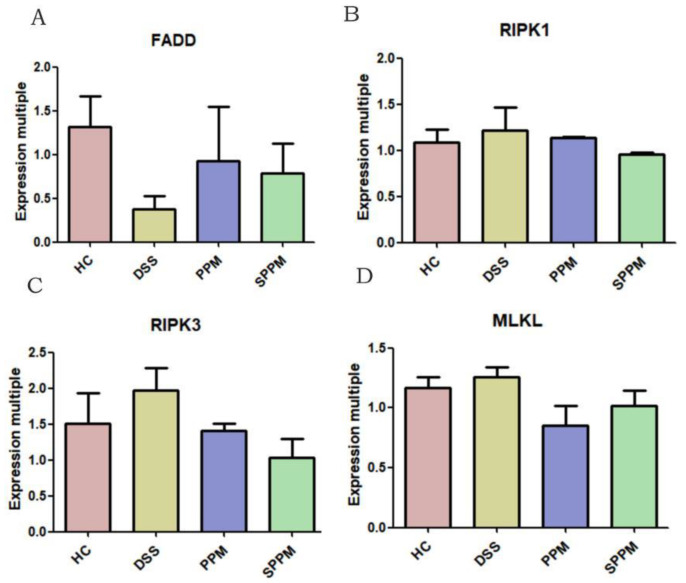
Effects of PPM60-III and SPPM60-III on genes related to necroptosis in mice. (**A**) *RIPK3*; (**B**) *RIPK1*; (**C**) *MLKL*; (**D**) *FADD*.

As shown in Figure 6, the PCR results showed that DSS could reduce the relative expression of FADD mRNA and increase the relative expressions of RIPK1, RIPK3, and MLKL mRNA. However, PPM60-III and SPPM60-III have the opposite effects, but there was no significant difference.

**Figure 7:**
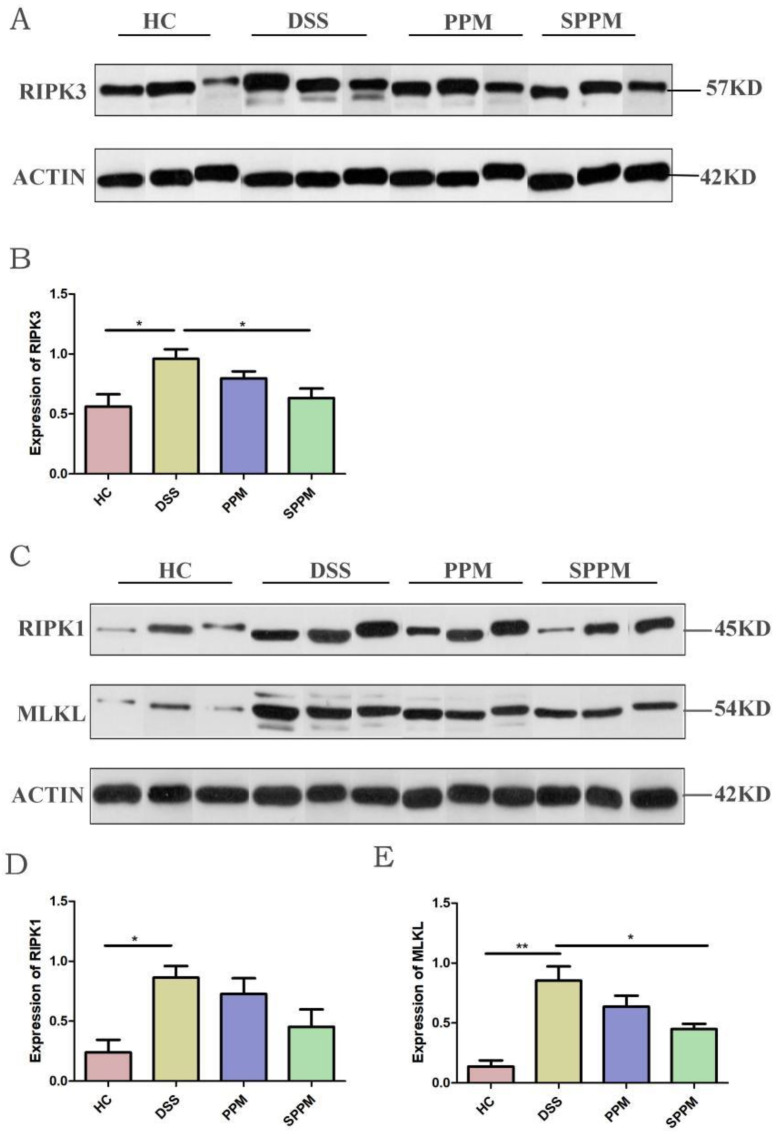
Expression of proteins related to necroptosis in mice by PPM60-III and SPPM60-III. (**A**) Protein expression bands of RIPK3; (**B**) quantitative analysis of RIPK3; (**C**) protein expression bands of RIPK1 and MLKL; (**D**) quantitative analysis of RIPK1; (**E**) quantitative analysis of MLKL. * *p* < 0.05, ** *p* < 0.01.

Western blotting showed that, compared with the HC group, the DSS group could significantly increase the protein expressions of RIPK1 (*p* < 0.05), RIPK3 (*p* < 0.05), and MLKL (*p* < 0.01) in colon tissue, while the three proteins in the PPM group and SPPM group had different degrees of recovery. The SPPM group had the best recovery effect and had a significant difference in reducing RIPK3 and MLKL proteins (*p* < 0.05) (Figure 7). The results showed that SPPM60-III could significantly improve the expressions of RIPK3 and MLKL proteins and PPM60-III could also regulate the expressions of three proteins, but there was no significant difference.

**Figure 8:**
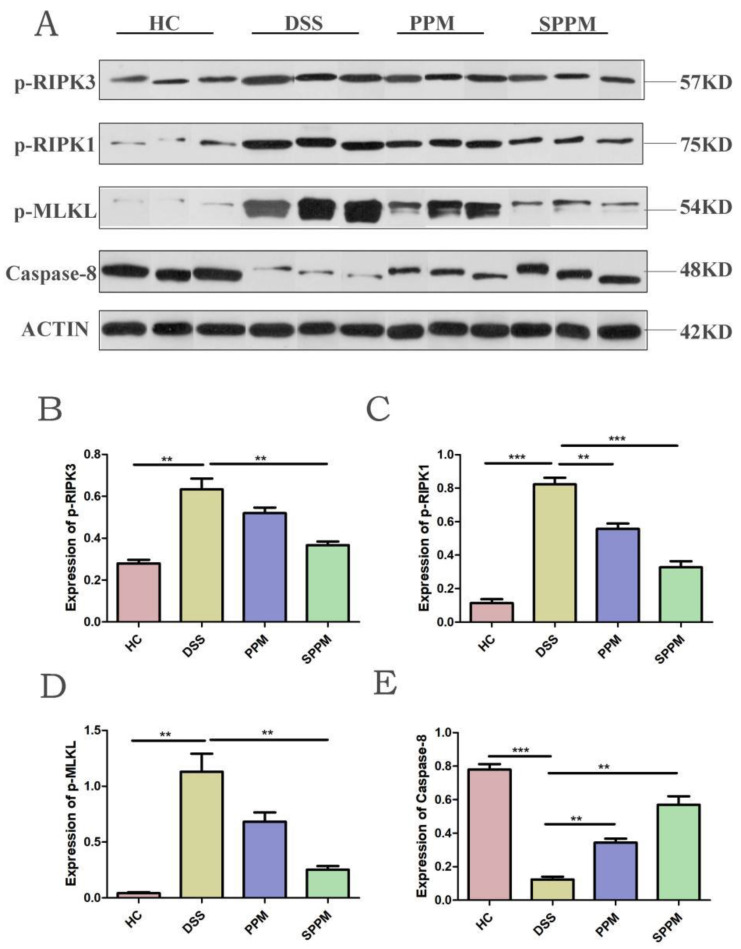
Expression of PPM60-III and SPPM60-III on phosphorylation of proteins associated with necroptosis in mice. (**A**) Protein expression bands of p-RIPK1, p-RIPK3, p-MLKL, and Caspase-8; (**B**) quantitative analysis of p-RIPK3; (**C**) quantitative analysis of p-RIPK1; (**D**) quantitative analysis of p-MLKL; (**E**) quantitative analysis of Caspase-8. ** *p* < 0.01, *** *p* < 0.001.

Phosphorylation of RIPK1, RIPK3, and MLKL is necessary in the necroptosis pathway. Therefore, Western blotting of three phosphorylated proteins was performed. The results also showed that, compared with the HC group, the expressions of p-RIPK3 (*p* < 0.01), p-RIPK1 (*p* < 0.001), and p-MLKL (*p* < 0.01) in the DSS group were significantly increased, while the expressions of the three proteins in the PPM group and SPPM group were decreased to varying degrees. The three proteins in the SPPM group showed a significant decreasing trend, while only p-RIPK1 in the PPM group was significant (*p* < 0.01). Meanwhile, the expression of Caspase-8 was inhibited in the DSS group, while the expression of Caspase-8 was significantly increased in the PPM group and SPPM group (*p* < 0.05), and the effect was better in the SPPM group (Figure 8).

## 3. Discussion

In the previous works of our laboratory, it was confirmed that pine pollen polysaccharide has anti-tumor and immune regulation functions. In addition, it also has other physiological activities, including regulating intestinal mucosal immunity [28,29], anti-virus [30], anti-oxidation, and protecting liver damage [31].

In this experiment, the classical UC mice model was established by inducing DSS to observe the effects of PPM60-III and SPPM60-III on the symptoms of UC mice.

The results showed that PPM60-III and SPPM60-III could significantly improve the pathological damage of the colon in mice, improve the weight loss caused by DSS-induced inflammation in mice, and significantly reduce the DAI score. The HE staining results showed that the colon injury of mice was alleviated after PPM60-III and SPPM60-III treatment. The results of ELISA experiment showed that PPM60-III and SPPM60-III could reduce the levels of pro-inflammatory cytokines IL-6, IL-18, IL-1β, and TNF-α in mice; increase the level of anti-inflammatory cytokines IL-10; and reduce the protein expressions of inflammatory-related enzymes COX-2 and iNOS. Overall, SPPM60-III has better effects on improving symptoms in mice. Studies have shown that the polysaccharide from *Potentilla anserina* L has an obviously anti-hypoxia activity, and it could effectively reduce lung water content, meliorate the histopathological damage of lung tissue, lessen the expression of pro-inflammatory cytokines, and inhibit the activity of oxidative stress [32]. *Echinacea purpurea* polysaccharide has strong antioxidant capacity to reduce the levels of inflammatory cytokines in both mouse serum and liver. Moreover, *Echinacea purpurea* polysaccharide maintained the integrity of barrier function by upregulating the expression of ileal tight junction proteins, thereby alleviating hepatic inflammation and damage [33]. *Turbinaria ornata* derived sulfated polysaccharide reduced the mRNA levels of *IL-6* and increased *IL-10* levels in the heart tissue, substantiating its anti-inflammatory potency [34]. Sulfated *Ganoderma applanatum* polysaccharide was homogeneous with a sulfur content of 7.8%. It exhibited hepatoprotective effects by reducing the histopathological damages, improving the anti-oxidative and anti-inflammatory properties [35]. Our results were similar to those of previous studies, suggesting that PPM60-III and SPPM60-III could improve inflammatory symptoms in mice to a certain extent.

The proposed and most accepted mechanism by which DSS induces intestinal inflammation results in the disruption of the intestinal epithelial monolayer lining, leading to the entry of luminal bacteria and associated antigens into the mucosa and allowing the dissemination of proinflammatory intestinal contents into underlying tissue [36]. The functional obstacle of the intestinal tight junction barrier is due to pro-inflammatory factors such as IFN-γ, TNF-α, and IL-1β, which is a potential mechanism leading to intestinal barrier defects [37]. The integrity of the intestinal mucosal barrier is maintained by tight junction proteins; when the intestinal mucosal barrier was damaged, the expressions of tight junction proteins such as ZO-1, Occludin, and Claudin-1 decreased [38]. Polysaccharides also play a role in regulating tight junction protein expressions and maintaining the intestinal mucosal barrier to treat inflammation. *Ficus carica* polysaccharides protected the goblet cells, elevated the expression of tight junction protein Claudin-1, and suppressed the formation of cytokines including TNF- and IL-1β [39]. In addition, some scholars demonstrated that *Pinus massoniana* pollen polysaccharides mitigated lipopolysaccharide (LPS)-induced intestinal epithelial damage in Caco2 monolayer cells [40]. In this experiment, TEM showed that PPM60-III and SPPM60-III could restore the tight connection between colon epithelial cells and intestinal villi in mice. Western blotting showed that PPM60-III and SPPM60-III could significantly increase the protein expressions of ZO-1, Claudin-1, and Occludin in the colon epithelium of mice. The immunofluorescence results were consistent with the Western blotting results. The results in vivo were consistent with those in vitro [40]. The results showed that PPM60-III and SPPM60-III could reduce the damage of the colon barrier and restore tight junctions.

Polysaccharides also play an important role in regulating RIPK3-dependent necroptosis. Dark tea polysaccharide had a certain therapeutic effect on sepsis mice, which may be concerned with the inhibition of macrophage necroptosis induced by LPS [41]. *Lentinan edodes* polysaccharide could reduce the relative expression levels of *RIPK1*, *RIPK3*, *MLKL*, and *FADD* mRNA to regulate the necroptosis signaling pathway of piglets [42].

Studies have found that UC is closely related to RIPK3-dependent necroptosis. RIPK3 inhibitor ameliorates the severity of experimental colitis and reduces inflammation through the inhibition of the inflammatory response and necroptosis and supports RIPK3-targeting substances for the treatment of UC [43]. Activation of the RIPK3-p-MLKL axis increases the production of inflammatory molecules and alters the intestinal mucosa permeability [44]. During necroptosis, RIPK1 and RIPK3 interact with each other, resulting in the formation of the functional heterodimer complex; this complex promotes oligomerization of MLKL by phosphorylating it. The oligomeric form of MLKL translocates towards the plasma membrane from cytosol, resulting in the formation of the pore, causing an inflammatory response [22].

In this study, RIPK3-dependent necroptosis was used to study the therapeutic effects of PPM60-III and SPPM60-III on UC mice. The results showed that PPM60-III and SPPM60-III could reduce the expressions of *RIPK1*, *RIPK3*, and *MLKL* genes and increase the expression of *FADD* genes. In addition, Western blotting also showed that PPM60-III and SPPM60-III could significantly reduce the protein expressions of p-RIPK1, p-RIPK3, and p-MLKL and increase the protein expression of Caspase-8. The results showed that PPM60-III and SPPM60-III participated in regulating RIPK3-dependent necroptosis. These results are consistent with those of GUO and ZHANG et al. [23]. These results suggested that PPM60-III and SPPM60-III may have therapeutic effects on UC mice by regulating RIPK3-dependent necroptosis.

In summary, the effects of PPM60-III and SPPM60-III on tight junctions and RIPK3-dependent necroptosis in UC mice were studied. The results showed that PPM60-III and SPPM60-III could act on the RIPK3-dependent necroptosis pathway in UC mice, thereby reducing the phosphorylation expressions of proteins related to the pathway and inhibiting the production of pro-inflammatory factors. Inhibition of inflammatory factors increased tight junction protein expressions, restored tight junctions, and relieved colitis symptoms in UC mice. From all of the results, SPPM60-III was more effective than PPM60-III. The purpose of this study was to explore and find new natural drugs for the treatment of ulcerative colitis and provide a new reference direction for the treatment of UC. These studies may be helpful in food development and research. Therefore, pine pollen polysaccharides and esterified polysaccharides could be developed as effective food and health care products to prevent UC and restore intestinal epithelial injury. However, its specific mechanism and target are still unclear and need to be further studied.

## 4. Materials and Methods

### 4.1. Materials and Reagents

Broken wall of yunnan pine pollen (obtained from Yantai New Era Health Industry Co., Ltd, Yantai, China); dextran sulfate sodium salt (DSS, 36,000-50,000 Da) was purchased from MP Biomedicals (Santa Ana, CA, USA); the ELISA kits of IL-6, IL-10, IL-1β, IL-18, and TNF-α (obtained from MultiSciences (Lianke) Biotech Co., Ltd, Hangzhou, China); antibodies against COX-2, iNOS, ZO-1, Occludin, Claudin-1, RIPK1, RIPK3, MLKL, Caspase-8, p-RIPK1, p-RIPK3, p-MLKL, and immunofluorescence secondary antibody (obtained from Wuhan Servicebio Biological Co., Ltd, Wuhan, China); DAPI (obtained from Wuhan Servicebio Biological Co., Ltd, Wuhan, China).

### 4.2. Extraction, Separation, and Purity Detection of Polysaccharides from Pine Pollen of Pinus yunnanensis

The extraction method of polysaccharide from pollen of *Pinus yunnanensis* was based on the previous extraction process in the same laboratory. Broken masson pine pollen was provided by the Yantai New Era Health Industry Company (broken rate > 95%). Broken masson pine pollen polysaccharides were extracted using a water extract-ethanol precipitation method, with the coarse polysaccharides (PPM60) obtained by ethanol precipitation with 60% ethanol. Proteins were removed using the trichloroacetic acid precipitation method. PPM6-III was then purified from PPM60 by Sephacryl S-400HR. The content of polysaccharides was determined by the phenol sulphuric acid method and the OD value was measured at 490 nm. The curve was drawn by taking the number of test tubes collected as abscissa and the OD value as ordinate [27].

### 4.3. Sulfation of Polysaccharides from Pine Pollen

A sulfate derivative, named SPPM6-III, was then obtained using chlorosulfonic acid pyridine. The barium sulfate turbidity method was used to assess sulfur content, with the degree of substitution then calculated. The standard curve was first drawn with sulfate concentration as abscissa and OD value as ordinate. The sulfate content was calculated by substitution. The OD value of sulfated polysaccharide was substituted into the standard curve and the substitution degree was calculated by substituting the substitution degree formula of polysaccharide. The calculation formula of the substitution degree of polysaccharide is as follows:

DS = (162 × S)/(32 − 102 × S)(1)

Polysaccharide powder was dried prior to tableting with KBr powder. IS50 Fourier transform infrared (FT-IR) spectroscopy (Thermo, MA, USA) was performed to investigate the FT-IR spectrum, detected in the frequency range of 4000–400 cm^-1^ [27].

### 4.4. Animals and Model

Healthy male C57BL/6 mice (6 weeks of age, 18–22 g) were purchased from the Beijing Weitong Lihua Experimental Animal Technology Co., Ltd, Beijing, China. Standard conditions of 22 ± 2 °C, 40–60% humidity, and a 12 h light/dark cycle were maintained. All mice were adapted at least 7 days before they were used in experiments. All experiments were carried out according to the Guidelines for the Care and Use of Animals, and all experiment procedures were approved by the Laboratory Animal Center of Shandong Normal University Committee (Approved No. AEECSDNU2021085).

According to the results of the preliminary experiment, three doses were designed and, finally, PPM60-III and SPPM60-III were both 100 mg/kg/day.

All animals were randomly assigned to four groups and received treatment for one continuous week: (a) the HC group, the control group, treated with drinking water; (b) the DSS group, treated with both drinking water and 3% DSS for 7 days; (c) the PPM group, drinking 3% DSS and administrated with 100 mg/kg/day PPM60-III for 7 days; (d) the SPPM group, drinking 3% DSS and administrated with 100 mg/kg/day SPPM60-III for 7 days; with eight mice in each group. The HC group and DSS group were given 0.2 mL water by gastric lavage from first day to seventh day. The PPM group and SPPM group were given 0.2 mL PPM60-III (100 mg/kg/day) and SPPM60-III (100 mg/kg/day). The concentration of DSS and the dose of PPM60-III and SPPM60-III were determined according to the results of the preliminary experiment. The weight and feces of mice were recorded every day. Seven days later, after being fasted for 24 h, mice were anesthetized and sacrificed, then the colons, feces, and blood were collected for further analysis.

### 4.5. Assessment of Disease Activity Index (DAI)

The DAI is a comprehensive score based on the percentage of weight loss, stool consistency, and stool bleeding of the patient (the affected animal). The DAI score is the average of the three results.

According to Table 1, the DAI of each mouse is obtained to evaluate the disease activity.

The disease activity index = (combined score of weight loss, stool consistency and bleeding)/3 [45].

### 4.6. Colon Length Analysis

At the end of the experiments, the mice were killed and the colon lengths were determined. Parts of the colon tissues were fixed with 4% paraformaldehyde and paraffin embedded for H&E staining and immunofluorescence staining. The rest of the colon tissues were stored at -80 °C for Western blotting and other experiments.

### 4.7. H&E Staining

A small section of the colon was taken from each group, after being fixed with 4% paraformaldehyde for 48 h, and the colon tissues were embedded with paraffin. Tissue sections (5 m) were prepared. After stained with hematoxylin for 3–5 min, sections were stained with eosin for 5 min. Images were captured by a Nikon Eclipse E100 microscope (Nikon, TKY, Japan).

### 4.8. Electron Microscope Analysis

To examine the ultrastructure of the colon, the electron microscope analysis was carried out in colonic tissues. Briefly, colon tissues were fixed in 2.5% glutaraldehyde (pH 7.4) and 1% osmium tetraoxide (pH 7.4). After conventional embedding, tissues were sliced into 60–70 nm and stained with uranyl acetate and lead citrate. Finally, the ultrastructures of the colon tissues were examined and photographed using a HT-7800 transmission electron microscope (Hitachi Corporation, Beijing, China).

### 4.9. Expression of Inflammatory Cytokines by ELISA

Serum samples were obtained by centrifugation (3000 rpm, 10 min) and stored at −80 °C. The serum levels of IL-10, TNF-α, IL-1β, IL-18, and IL-6 were determined with ELISA kits following the manufacturers’ instructions.

### 4.10. Western Blotting

Here, 50 mg tissues were dissolved in 0.5 mL protein lysis buffer and ground on ice to extract proteins. Protein concentrations were quantified using BCA protein assay kits. The samples were denatured at 95 °C for 15 min and then isolated with 5% SDS-PAGE. Subsequently, proteins were transferred onto PVDF membranes and blocked with 5% nonfat milk followed by incubation with primary antibodies (-actin (1:1000), COX-2 (1:1000), iNOS (1:1000), ZO-1 (1:1000), Occludin (1:1000), Claudin-1 (1:1000), RIPK1 (1:1000), RIPK3 (1:1000), MLKL (1:1000), Caspase-8 (1:1000), p-MLKL (1:1000), p-RIPK1 (1:1000), and p-RIPK (1:1000). Then, the membranes were immunoblotted with several primary antibodies overnight at 4 °C. After washing with TBST three times, the membranes were incubated with a 1:5000 diluted secondary antibody for 1 h at room temperature before they were washed with TBST three times. After washing the film, the G2014 ECL (Servicebio) method was used to develop and fix the film and the exposure conditions were adjusted. The gel image was analyzed and the film was scanned and archived. Photoshop was arranged and color was removed, and the Alpha software processing system was used to analyze the optical density value of the target band [46].

### 4.11. Immunofluorescence Staining

Immunofluorescence analysis was performed on colon tissues. A paraffin section of the colon tissue of mice was made, including sampling, fixation, dehydration, dipping wax, embedding, and sectioning. The tissue slices were placed into EDTA antigen repair solution for repair. After blocking with 3% bovine serum albumin (BSA), the samples were incubated with primary antibodies overnight at 4 °C. The next day, the fluorescence secondary antibody was added and incubated for 2 h at RT, and DAPI reagent was used to stain the nuclei. Finally, samples were imaged with a Nikon Eclipse C1 fluorescence microscope (Nikon, TKY, Japan). Then, the number of positive cells and the total number of cells in each section were quantified using Indica Labs—HighPlex FL V3.1.0 module in Halov3.0.311.314 analysis software, and the positive rate was calculated (100%) [47].

### 4.12. RNA Preparation and Quantitative Real-Time PCR Analysis

About 100 mg of colon tissue was taken and added into the homogenization tube containing RNA extract, and it was centrifuged at 12,000 rpm for 10 min to obtain the supernatant.

Total RNA was extracted from tissues, then Nanodrop 2000 was used to detect the concentration and purity of RNA, the RNA with too high a concentration was diluted to an appropriate proportion so that the final concentration was 100–500 ng/μL, and the RNA was reverse-transcribed into cDNA using the RT-PCR kit. Next, the RT-qPCR was performed. The 2^-ΔΔCt^ method was used to analyze the expression levels of target genes and the -actin was used as an internal control. Primers were designed according to mouse sequences. The primer sequences are shown in Table 2, and were synthesized by Wuhan Servicebio Biotechnology Co., Ltd, Wuhan, China [47].

### 4.13. Statistical Analysis

All statistical analyses were performed by SPSS software and Graph-pad prism 7 software, T-test methods were used in the statistical analysis, and the results were presented as the mean ± SEM (standard error of the mean). All experiments were repeated at least three times.

## Figures and Tables

**Table 1:** DAI scoring criteria.

DAI Score	Weight Loss	Stool Consistency	Occult/Gross Bleeding
0 1	0 1~5%	Normal	Normal
2 3	5~10% 10~15%	Loose	Guiac (+)
4	>15%	Diarrhoea	Gross bleeding

Normal stools = well-formed pellets; loose = pasty stools that do not stick to the anus; diarrhoea = liquid stools that stick to the anus.

**Table 2:** Primer sequences for real-time quantitative PC.

	Forward5′-3′	Reveser5′-3′
GAPDH	CCTCGTCCCGTAGACAAAATG	TGAGGTCAATGAAGGGGTCGT
FADD	CGCGTGAGCAAACGAAAGC	CACACAATGTCAAATGCCACC
RIPK1	AGAAGAAGGGAACTATTCGCTGG	CATCTATCTGGGTCTTTAGCACG
RIPK3	CAGTGGGACTTCGTGTCCG	CAAGCTGTAGGTAGCACATC
MLKL	TTAGGCCAGCTCATCTATGAACA	TGCACACGGTTTCCTAGACG

## Data Availability

Not applicable.

## Sample Availability

Samples of the compounds not available are available from the authors.
